# Neural Tuning Size in a Model of Primate Visual Processing Accounts for Three Key Markers of Holistic Face Processing

**DOI:** 10.1371/journal.pone.0150980

**Published:** 2016-03-17

**Authors:** Cheston Tan, Tomaso Poggio

**Affiliations:** 1 McGovern Institute for Brain Research, Massachusetts Institute of Technology, Cambridge, Massachusetts, United States of America; 2 Department of Brain and Cognitive Sciences, Massachusetts Institute of Technology, Cambridge, Massachusetts, United States of America; 3 Visual Computing Department, Institute for Infocomm Research, Singapore, Republic of Singapore; Harvard Medical School, UNITED STATES

## Abstract

Faces are an important and unique class of visual stimuli, and have been of interest to neuroscientists for many years. Faces are known to elicit certain characteristic behavioral markers, collectively labeled “holistic processing”, while non-face objects are not processed holistically. However, little is known about the underlying neural mechanisms. The main aim of this computational simulation work is to investigate the neural mechanisms that make face processing holistic. Using a model of primate visual processing, we show that a single key factor, “neural tuning size”, is able to account for three important markers of holistic face processing: the Composite Face Effect (CFE), Face Inversion Effect (FIE) and Whole-Part Effect (WPE). Our proof-of-principle specifies the precise neurophysiological property that corresponds to the poorly-understood notion of holism, and shows that this one neural property controls three classic behavioral markers of holism. Our work is consistent with neurophysiological evidence, and makes further testable predictions. Overall, we provide a parsimonious account of holistic face processing, connecting computation, behavior and neurophysiology.

## Introduction

Faces are an important class of visual stimuli with unique significance, and face processing is a longstanding topic of active study within neuroscience (e.g. [1–4]). Faces are ubiquitous throughout a person’s life, and face recognition is important for daily social interaction. An important way in which visual processing of faces and non-face objects differs, is that faces have been found to elicit certain characteristic behavioral markers. These have been explained qualitatively through the loose notion of “holistic processing”. However, the exact nature of holism is poorly understood, with multiple definitions, interpretations and putative mechanisms [5–7].

Importantly, little is known about the neural mechanisms underlying holistic face processing. For face processing in general by the primate and human visual systems, multiple neural correlates and signatures are known, but the actual neural computations, particularly for holistic processing, are still a mystery. Thus, we seek to explain precisely what gives rise to holistic face processing in terms of neural computation mechanisms.

We performed computational simulations using a model from the family of neurobiologically-plausible visual recognition models [8–12]. The model has four layers. The orientation-selective lower two layers (*S1* and *C1*) simulate V1 simple and complex cells tuned to various orientations, at different locations and spatial frequency scales. Above *C1*, the next layer (*S2*) contains model neurons that learn templates from face images during an unsupervised template-learning process performed prior to normal model operation. The *S2* layer responses are the outputs from matching the learnt templates to the incoming pattern of *C1* responses generated by a new image being processed. Finally, each *C2* model neuron computes the maximum response among *S2* model neurons with identical templates but receptive fields at different spatial locations and scales. Hence, *C2* responses are selective for stimulus appearance, but invariant to location and scale. (See Materials and Methods for model details.)

Using our model, we show that a single factor–“neural tuning size”–is able to account for three classic behavioral phenomena that are characteristic of face processing, namely the Composite Face Effect (CFE), Face Inversion Effect (FIE) and Whole-Part Effect (WPE) (respectively: [13], [14], [15]). Tuning size controls whether processing style is “face-like” or “object-like”, as gauged by these three important markers of holism.

We define “neural tuning size” as the size of the *S2* template that specifies the optimal stimulus of each *C2* model neuron, i.e. the number of C1 model neurons that contribute to each *S2* template. Importantly, this definition of tuning size is in terms of the proportion of a whole face covered by a template, which may or may not be related to number of pixels or degrees of visual angle. To various extents, there exists invariance to image scale in our model, as well as in the human and primate visual systems. Therefore, a particular tuning size (e.g. half-a-face) can correspond to a range of sizes in pixels or degrees of visual angle. We primarily compared large tuning size (covering multiple face parts, but less than half the whole face) with small tuning size (roughly the size of an eye or nose).

Our computational proof-of-principle specifies the precise neural tuning property that corresponds to the poorly-understood notion of holistic face processing, and shows that a computational realization of this neural property actually produces the relevant psychophysical behavior. Our work also makes testable predictions for neurophysiology and psychophysics.

## Results

Our simulation results show that when tuning size is large, even though each template covers less than half a whole face, three classic markers of holistic processing (the CFE, FIE and WPE) are produced. Conversely, a single change–reduction of tuning size–leads to “object-like” non-holistic processing. This strongly suggests that tuning size is a key factor underlying holistic processing. (See Materials and Methods for simulation details.)

**Fig 1:**
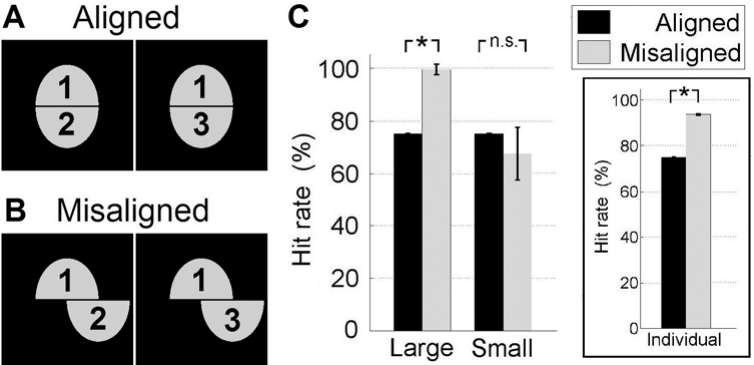
Tuning size accounts for the Composite Face Effect (CFE). (A) Schematic for aligned composite faces in *“same”* trials. Top halves are identical, while bottom halves are different. People sometimes incorrectly perceive the two identical top halves as different. (B) Schematic for misaligned composite faces. Human judgement of the top halves (as being identical) is significantly more accurate for misaligned than aligned composites. (C) The CFE is produced by *C2* model neurons with large–but not small–tuning size. (Inset) Each individual *C2* model neuron with large tuning size can produce the CFE by itself. Error bars: SEM.

The Composite Face Effect (CFE) [13] is the phenomenon whereby two identical top halves are sometimes incorrectly perceived as different, when paired with different bottom halves (Fig 1A). This effect is ostensibly due to the top and bottom halves of each composite being perceived “holistically” (together as a whole) when aligned, despite instructions to ignore the bottom halves. Perception of the top halves as being identical is more accurate when the halves are misaligned (Fig 1B). Crucially, this effect occurs only for faces, and is commonly taken as evidence that face and object processing are qualitatively different [16–18].

Behaviorally, the standard experimental procedure is that on each trial, two composites (either with *same* or *different* top halves) are presented. Human subjects are told to ignore the bottom halves (which are always different) and determine if the top halves are same or different. Only the *same* trials, i.e. with identical top halves, are analyzed [17]. The CFE is defined as a higher hit-rate (i.e. accuracy on the *same* trials) for misaligned than aligned composites.

Our simulations show that large tuning size produces the CFE, but not small tuning size (Fig 1C, *Large*: p = 0.001, *Small*: p = 0.85, paired bootstrap test comparing *Misaligned* and *Aligned* hit-rates, 1000 resamples). The CFE is also found using each individual *C2* model neuron with large tuning size by itself (Fig 1C inset, p<0.0001, Wilcoxon signed-rank test, n = 1000 *C2* model neurons), even though tuning size is less than half the whole face, and there is no overlap between receptive fields to speak of. Conversely, even though the set of 1000 *C2* model neurons with small tuning size collectively cover the whole face many times over, they do not produce the CFE (Fig 1C, *Small* condition).

**Fig 2:**
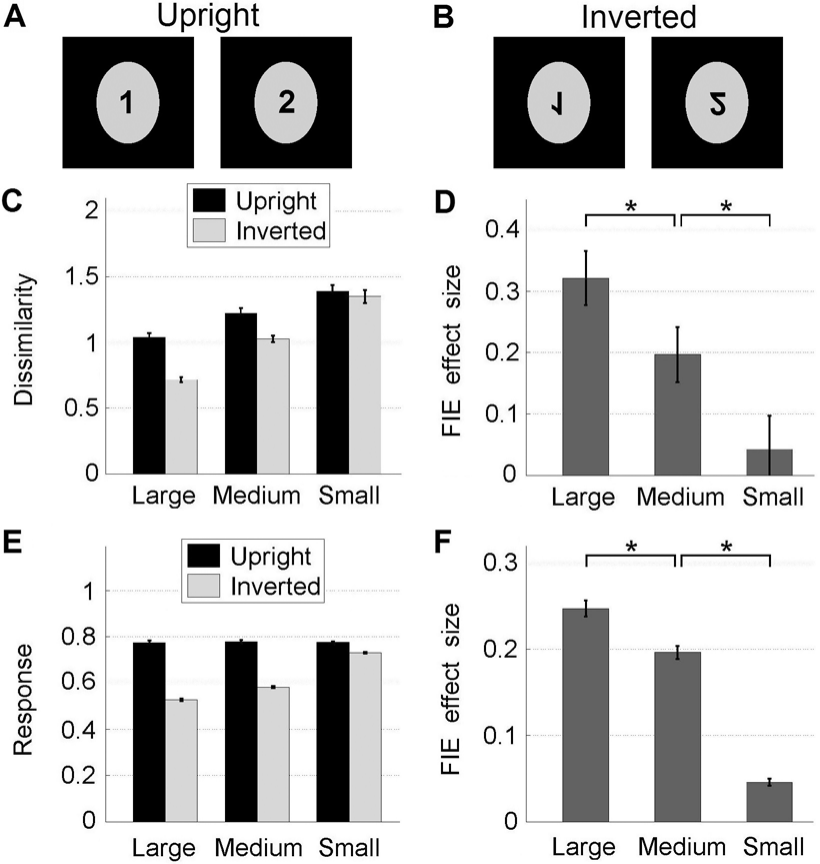
Tuning size accounts for the Face Inversion Effect (FIE) at the behavioral and neural levels. (A-B) Schematic for the FIE: dissimilarity between faces is more apparent for upright (A) than inverted (B) faces. (C) Mean dissimilarities between patterns of *C2* responses to two different faces, for all possible pairs of faces. (D) Behavioral FIE effect size (upright dissimilarity–inverted dissimilarity in (C)) varies with tuning size. (E) Results for the neural-level FIE, i.e. mean individual *C2* model neuron responses to each face (as opposed to dissimilarities between sets of neural responses to pairs of faces, in (C)) for upright vs. inverted faces. (F) Neural-level FIE effect size (upright response–inverted response in (E)) varies with tuning size. Error bars: SEM.

Tuning size also accounts for another key face-specific phenomenon, the Face Inversion Effect (FIE) [14], whereby upside-down inversion disrupts face processing significantly more than object processing (Fig 2A and 2B). Fig 2C shows the mean dissimilarity (euclidean distance between two sets of *C2* layer responses) within each of all the possible 1225 (i.e. ^50^C_2_) pairs of faces. Fig 2D shows the mean “behavioral” FIE effect size (i.e. upright–inverted dissimilarities shown in Fig 2C). When tuning size is reduced, the behavioral effect of inversion is also significantly reduced, akin to the processing style becoming “object-like” (Fig 2D: *Large>Medium*: p = 0.023, *Medium>Small*: p = 0.012, *Large>Small*: p = 0.0002, paired bootstrap test, 10000 resamples).

Inversion also reduces the mean response of each individual *C2* model neuron, accounting for the neural basis of the Face Inversion Effect [19]. Fig 2E shows the mean neural response, averaged over all 1000 *C2* model neurons’ responses to all 50 faces. Importantly, tuning size also controls the magnitude of the FIE at the individual-neuron level (Fig 2F, *Large>Medium*: p<0.0001, *Medium>Small*: p<0.0001, Wilcoxon signed-rank test, n = 50 faces). For the FIE, the results from the medium tuning size condition in both Fig 2D and 2F reinforce the finding that according to our model, it is tuning size that controls FIE effect size.

**Fig 3:**
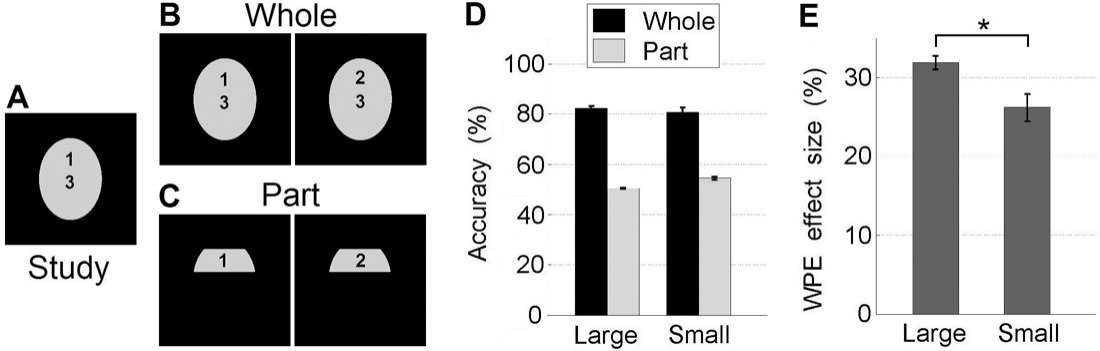
Tuning size accounts for the Whole-Part Effect (WPE). (A) Schematic for Study face. (B) Schematic for Whole condition: two choices (differing only in the eye region) are presented, to be matched to the study face from memory. (C) Schematic for Part condition: two choices (eye regions cropped from Whole condition) to be matched to the study face. (D) Mean accuracies for choosing correctly the Study face. (E) WPE effect size (Whole accuracy–Part accuracy) varies with tuning size. Error bars: SEM.

Finally, tuning size also accounts for the Whole-Part Effect (WPE) [15], a “gold-standard” test of holistic processing, like the CFE [17–18]. In WPE studies, subjects are first presented a study face (Fig 3A) to memorize. At test, in the *Whole* condition, subjects are presented with the study face and another face that differs only in a localized region (e.g. eye region), and have to recall and choose the study face (Fig 3B). In the *Part* condition, the differing localized regions in the *Whole* condition are cropped and presented instead (Fig 3C). Empirically, human subjects are significantly more accurate at choosing the study face in the *Whole* than *Part* condition; this phenomenon is termed the Whole-Part Effect. This effect is highly significant for faces, but non-significant or significantly smaller for non-faces [17].

Our results show that tuning size can account for the WPE. To simulate subjects choosing between the two test faces, the face with the smaller dissimilarity (Euclidean distance between two sets of *C2* layer responses) to the study face was picked. Fig 3D shows the mean accuracies when using *Large* and *Small* tuning sizes. Importantly, reduction in tuning size results in a significantly smaller WPE effect size (i.e. *Whole*–*Part* accuracy in Fig 3D), akin to more “object-like” processing (Fig 3E, *Large>Small*: p = 0.001, paired bootstrap test, 1000 resamples).

Overall, our results show that tuning size can account for the CFE, FIE and WPE. In all these three markers of face-specific holistic processing, large tuning size reproduces the effect, while reduction to small tuning size results in the abolishment of the effect (CFE) or a significantly smaller effect size (FIE and WPE).

## Discussion

Holism is poorly-understood, with multiple definitions, interpretations and putative mechanisms [5–7]. Large tuning size is not the only understanding of holism that has been proposed. “Processed as a unified whole”, “obligatory processing of all parts” and “represented as a unitary, undecomposed whole” are some qualitative definitions of holism. However, these are imprecise descriptions that do not specify the exact computations responsible.

Here, we offered a precise operational definition, termed “large tuning size” for conciseness. The idea bears similarities to theoretical proposals by others, (e.g. [16, 20–23]. Crucially, we show that the realization of this definition using a quantitative computational model actually produces classic markers of holistic processing–and also its abolishment or reduction when tuning size is small.

This is the first time any single model has accounted for all these three key markers. Other variations of the CFE have been demonstrated [12, 24], while variations of the WPE have been demonstrated [25, 26]. The FIE has been demonstrated by many models. Our work not only accounts for all the three effects, but also specifically identifies the mechanism responsible and directly shows that manipulation of this mechanism controls these effects.

In our model, even though the set of 1000 small templates collectively processes all parts of the face many times over (1000 x 4x4 / 17x22 = 42.8), they do not display holism, which suggests that “obligatory processing of all parts” per se is not crucial. Additionally, each individual large template covers less than half a whole face (12x12 / 17x22 = 0.39), yet each template by itself can demonstrate holism (Fig 1C inset), suggesting that a “unified whole” is not necessary, nor is overlap between templates. Our large-template model “decomposes” a face into a collection of *C2* responses, yet demonstrates holism, suggesting “undecomposed” representations are not critical.

Tuning size is the sole change between conditions, suggesting that other factors (e.g. attention) may not be root causes of holism. Additionally, while detection and segmentation are important processes for face recognition, the absence of explicit mechanisms for these also suggest that they are also not key factors. Of course, these factors may modulate the size of holistic effects, even if they are not underlying causes of holism.

### Learning and expertise

The role of learning and expertise in holism is still unclear [17, 27–29]. Our current work does not specify how or why templates with large tuning size may come about–though there is some theoretical and empirical justification [30, 31]–and is therefore agnostic as to whether expertise or learning can result in holistic processing. It is also agnostic as to whether only faces could ever have category-selective neurons with large tuning size and therefore perform holistic processing.

However, what our model does indicate, is that large tuning size results in holistic processing. Conversely, it suggests that when processing is holistic (for faces or otherwise, whether due to innateness, development or expertise) large tuning size may be a mechanistic root cause. Our results do not deny or rule out the existence of face-selective neurons with small tuning size, or that some aspects of face processing could be non-holistic.

### Predictions

One important use of models is to make predictions. Our model predicts that in brain areas that process faces holistically, a biological neuron’s optimal stimulus (i.e. face image that causes the neuron to fire maximally) can be smaller than a whole face. Starting from individual face parts, increasingly larger contiguous face portions will be shown. Individual neuron response magnitudes should increase with size, and a significant number should saturate before the entire face is shown. This prediction has partially been shown by the finding that in macaque face patches MF/ML, neurons are tuned to at most four (out of seven) face parts [4]. On the surface, this empirical finding is seemingly difficult to reconcile with the notion of holistic (“as a whole”) processing, but our results show that tuning to literal wholes is not necessary for holistic processing.

Within holistic face areas, tuning size may vary [4], and our work predicts that measures of holism are graded (not binary), and correlate with measures of tuning size. Furthermore, different measures of holism should be correlated with each other, since we show that three classic markers can arise from the same mechanism.

A behavioral prediction is that controlling “largeness of processing” also controls holism. One way is to show faces in a gaze-contingent manner, revealing only pixels within a certain radius from fixation point. Subjects can look around freely, so the whole face is visible, just not all at once. We predict that with small apertures, only neurons with small tuning size will respond, so processing is non-holistic. Conversely, apertures that are large-but-not-whole should allow for holistic processing to happen.

## Materials and Methods

### Model

We used the HMAX model architecture [32, 33], part of a family of neurobiologically-inspired models that simulate hierarchical processing in the primate ventral visual cortex, reflecting the increase in neural tuning complexity and invariance up the hierarchy. The lowest layer in the hierarchy corresponds to orientation-selective V1 simple cells, while the highest layer corresponds to cells in inferotemporal cortex.

At all layers, each model neuron produces an output response between 0 and 1, where 1 signifies that the input matches the neuron’s optimal stimulus perfectly. The optimal stimulus could be an oriented Gabor at a specific location and scale (*S1* layer) or with some location and scale invariance (*C1*), or some portion of a specific face at some rough location and scale (*S2*) or at any location and scale (*C2*).

In response to each input image, the model produces 1000 *C2* model neuron responses as output. In all simulations, each condition used all and only these 1000 *C2* output responses, unless otherwise specified.

### Detailed operation and parameters

We used the HMAX implementation found at http://cbcl.mit.edu/jmutch/cns/ (specifically, the HMAX package within the CNS simulation software). Each input image (256x256 pixels) is downscaled to produce a multi-scale pyramid of images. We used 10 scales, with each scale downscaled by a factor of 2^(1/4) from the previous (larger) scale. At the *S1* layer, the multi-scale pyramid is convolved with Gabor filters (with default parameters) at 4 orientations. *S1* output responses are the normalized dot-product between the filter and the convolved region of the multi-scale pyramid. Going from *S1* to *C1*, each *C1* model neuron pools over 8x8 *S1* neurons from 2 adjacent scales, outputting the maximum value from the 128 values in this pooled region. This max-pooling region is shifted 3 steps (i.e. *S1* neurons) from one *C1* model neuron to its immediate neighbor. Going from *C1* to *S2*, the set of *C1* responses are convolved with *S2* templates (see next section). *S2* output responses are calculated as the similarity between the *S2* template and the corresponding region of *C1* responses. The similarity metric used was the gaussian radial basis function with sigma (width) parameter 1/3. This processing was repeated separately for each *S2* template, i.e. one template results in one pyramid of *S2* output responses. Going from *S2* to *C2*, each *C2* model neuron takes the maximum value over one entire pyramid of *S2* output responses, thus there are as many *C2* model neurons as *S2* templates i.e. 1000.

### Template learning (training)

Learning of *S2* templates simply means storing patterns of *C1* responses produced in response to some set of training images. This is the only point at which any learning or training is done in the entire process, and is done prior to any CFE/FIE/WPE simulations. Subsequently, during normal model operation as part of the simulations, these stored patterns of *C1* responses act as templates that the *C1* responses produced by new images are matched against. Training images consist of faces, thus *S2* and *C2* model neurons are face-selective, whereas *S1* and *C1* model neurons are pre-defined and fixed to be orientation selective. Following [32], for simplicity and to ensure roughly uniform coverage of all locations and scales, patterns of *C1* responses at randomly-chosen locations and scales were stored as *S2* templates. For each tuning size (see below), 20 *S2* templates were learnt from each of 50 training images of faces, giving 1000 *S2* templates for each tuning size. Training images were distinct from images used during the CFE/FIE/WPE simulations (see Stimuli description below).

### Tuning size

The critical independent variable in our simulations is “tuning size”. Large, medium and small tuning sizes correspond respectively to *S2* templates covering 12x12x4, 8x8x4 and 4x4x4 *C1* model neurons (where the third dimension is due to 4 orientations), all from the relatively coarse scale 7 (out of 9 spatial frequency scales that exist at the *C1* layer). At this scale, the entire face corresponds to 17x22x4 *C1* model neurons, therefore each small template is roughly the size of a face part (e.g. eye, nose), while each large template covers multiple face parts but less than half the whole face. Medium and small templates were defined as the central 8x8x4 and 4x4x4 regions of the large templates (12x12x4).

### Stimuli

The face images were derived from 100 frontal-view male faces belonging to a face database provided by the Max-Planck Institute for Biological Cybernetics in Tubingen, Germany [34]. The database can be found at http://faces.kyb.tuebingen.mpg.de/. Faces were downscaled by 25%, and then oval-cropped to remove outline and external features (e.g. hair). Entire images were 256x256 pixels, while faces were 80x120 pixels. Faces were normalized so that all had the same pixel-value statistics (mean and variance). Backgrounds were black. Odd-numbered faces were used for template-learning, even-numbered faces for normal operation. All faces were upright unless explicitly inverted.

### Bootstrap simulations

We used the bootstrap technique in our simulations. In each bootstrap resample (or run), the population of 1000 (unless otherwise stated) *C2* model neurons was uniformly sampled, with replacement. This sample was then used as described below in the simulation details and *Results* section (e.g. in the CFE simulations, used for computation of dissimilarity between composites). The p-values were computed as the proportion of resamples for which the test statistic was true under the null hypothesis. For example, for the CFE, the p-value is the proportion of resamples for which the *Misaligned* accuracy is not larger than the *Aligned* accuracy.

### Composite Face Effect (CFE) simulation details

Composites were constructed by pairing the top half of one face with the bottom half of a different face (with a two-pixel gap added). Twenty faces were used; these were chosen prior to all simulations. To simulate human subjects looking and attending to the top halves, bottom-half pixel values were multiplied by 0.1, and faces shifted downwards so that the top halves occupied the image center. To simulate subjects comparing composites, if the dissimilarity between composites (Euclidean distance between the two sets of *C2* layer responses) was below some threshold, the composites were considered *“same”*. For each condition (e.g. small tuning size), the threshold was independently set so that the aligned, upright hit-rate (i.e. accuracy on *“same”* trials) was as close to 75% as possible. Results are qualitatively robust to the threshold used.

### Face Inversion Effect (FIE) simulation details

In Fig 2C and 2D, for the large and medium tuning sizes, a random subset of *C2* model neurons (100 for large, 150 for medium) were used in each bootstrap run, instead of all 1000 *C2* model neurons. This was to compensate for the larger coverage areas for the large and medium tuning sizes. In Fig 2E and 2F, only *C2* model neurons with mean response to upright faces between 0.75 and 0.80 were considered, after which *C2* model neurons were randomly chosen so that all tuning sizes ultimately used the same number of *C2* model neurons. This was to control for otherwise different mean upright responses, and also avoid ceiling and floor effects.

### Whole-Part Effect (WPE) simulation details

Stimuli were constructed by blending the eye region of one face with the rest of a different face. Stimuli in the *Part* condition (Fig 3C) were produced by cropping out the eye region of stimuli in the *Whole* condition (Fig 3B). All possible trials (3-way combinations of first eye region, second eye region, rest of face) for twenty original faces (same faces as for CFE) were tested. To simulate human subjects looking and attending at the eye regions of the test faces, non-eye-region pixel values were multiplied by 0.5, and faces shifted downwards so that the eye regions occupied the center.
